# Association Between Depressive Symptoms and Physiological Risk Factors for Falls

**DOI:** 10.1155/jare/2687374

**Published:** 2026-06-29

**Authors:** Natalia Reynaldo Sampaio, Gabriela do Nascimento Candido, Ully Alexia Caproni Correa, Tais Gonçalves Soares, Silvia Lanziotti Azevedo da Silva, Juscelio Pereira da Silva, Leani Souza Maximo Pereira, Daniele Sirineu Pereira

**Affiliations:** ^1^ Graduate Program in Rehabilitation Sciences, Universidade Federal de Minas Gerais, Belo Horizonte, Brazil, ufmg.br; ^2^ Department of Physical Therapy, Centro Universitario de Belo Horizonte–Uni-BH, Belo Horizonte, Brazil; ^3^ Department of Colective Health, Universidade Federal de Juiz de Fora, Juiz de Fora, Brazil, ufjf.br; ^4^ Department of Physical Therapy, Universidade Federal de Minas Gerais, Belo Horizonte, Brazil, ufmg.br; ^5^ Rehabilitation Sciences Department, UNIFAL-MG, Alfenas, Brazil; ^6^ Department of Health Sciences, Faculdade de Ciencias Medicas de Minas Gerais, Belo Horizonte, Brazil

**Keywords:** aging, depressive symptoms, falls

## Abstract

**Objectives:**

Objective: To investigate the association between depressive symptoms and falls in community‐dwelling older adults.

**Methods:**

Methods: This is a cross‐sectional study with 278 participants aged 60 or older. Information on clinical and sociodemographic data were obtained from a structured interview, depressive symptoms were assessed with the Geriatric Depression Scale (GDS), falls in the previous year were self‐reported, and risk of falls was assessed with the Physiological Profile Assessment (PPA). Fallers and nonfallers were compared with Mann–Whitney and chi‐squared tests. A binary logistic model was used to investigate the association between falls and GDS score, followed by a binomial negative regression model to analyze the association of GDS score and the number of falls. Finally, groups of individuals screened positive or negative for depression were compared for risk of falls and each individual’s PPA test.

**Results:**

Discussion: Fallers presented more chronic conditions, polypharmacy, worse subjective health status and higher GDS scores. Individuals with higher GDS scores presented a higher chance of falling. The number of falls was not associated to the GDS score; however, there was an association between falls and the number of morbidities. Positive screening for depression was associated to a higher risk of falls according to the PPA, as well as to reaction time.

**Conclusion:**

Conclusion: Fallers presented more depressive symptoms, morbidities, polypharmacy, and worse subjective health status. Depressive symptoms do not act directly over the number of falls; nonetheless, those with depressive symptoms presented a higher chance of falling, which may be associated to slower reaction time. Psychological symptoms play an important role in falls, and should, therefore, be considered in identifying older people at risk and in developing prevention programs.

**4 key points**
• Depressive symptoms were not directly associated with the number of falls in this population.
• Those with depressive symptoms have slower reaction time when compared to their nondepressive counterparts.
• Slower reaction time may be associated with a higher risk of falling for older people with depressive symptoms.
• Chronic conditions and polypharmacy and subjective health status could be a risk factor for falls in older subjects.

## 1. Introduction

Falls are the leading cause of accidental injury and death in the older population [1]. Falls events occur in a third of the older adult population, and these events are associated with a range of adverse outcomes, including hospitalizations, increased disability, and reduced quality of life [2–6]. Falls are widely recognized as multifactorial events, resulting from the complex interaction of physiological, psychological, and environmental factors, many of which are influenced by the aging process.

Growing evidence suggests an association between depressive symptoms and falls; however, the underlying mechanisms involved in this relation are not well understood [7, 8]. Individuals with depressive symptoms may present abnormal motor behaviors [9–11], which may be expressed in alterations in gross motor activities, increased reaction time, gait unsteadiness, and inability to adjust movement speed [12–14]. Conversely, the occurrence of falls, subsequent fear of falling, and reduction in mobility may have a negative influence on depressive symptoms [15, 16], highlighting the potentially bidirectional nature of this relationship.

Depressive symptoms are highly prevalent amongst older people, affecting between 7.2% and 49% of those over the age of 65 worldwide [17, 18]. Even in the absence of a formal diagnosis of major depression, depressive symptomatology is associated to a higher risk of developing major depression, physical disabilities, clinical conditions, and higher use of medical services [19–21]. Moreover, it is worth mentioning that psychological conditions, such as the fear of falling, anxiety, and especially depressive symptoms, have therefore been increasingly investigated in the context of falls [7, 22–24].

In addition to depressive mood, other factors may contribute to falls in older people. Advanced age, polypharmacy, being female, and chronic conditions are frequently mentioned in the literature [25]. Many risk factors for falls are inherent to the aging process, such as muscle weakness, poor balance, impaired gait, and mobility and slower reaction time [26–29]. Importantly, the distribution and impact of those factors may differ between populations and healthcare contexts.

In the most recent guideline regarding falls prevention and management in older adults, experts suggested falls be considered under four different aspects, namely, predictive, preventative, personalized, and participatory [30]. This framework emphasizes early identification of fall risk factors, implementation of individualized prevention strategies based on patients’ clinical and social contexts, and active involvement of older adults in decision‐making processes.

While many studies have focused on falls as an outcome, there is a growing recognition of the importance of assessing modifiable risk factors—particularly those amenable to intervention—before falls occur in order to prevent downstream consequences such as mobility decline and loss of independence. In this regard, the assessment of sensory‐motor function may offer a valuable entry point for clinicians when assessing older adults with depressive symptoms who are at increased risk of falling.

Thus, the objective of this study was to investigate if depressive symptoms are associated with the occurrence of falls in this population. If so, our secondary objective was to establish which physiological measures might play a part in increasing the risk of falling in older adults with depressive symptoms.

## 2. Methods

Study design: This is an observational exploratory study with data collected transversally from the baseline of the project “Falls risk and mobility alterations in older people from the community: a longitudinal study”. The sample for this study was selected by convenience using an active search in the basic health units, senior activity centers, and in the university’s school clinic in the city of Alfenas, Brazil, in the year 2015. Every person over 60 years of age enrolled in the public health unit (Unidade Basica de Saude Pinheirinho) or senior activities in the center in the University’s area (UNATI–Universidade Aberta da Terceira Idade) and clinic was invited for an assessment, and all that accepted were trialed for inclusion and exclusion criteria. The study was approved by the ethics committee (CAAE: 49987915.4.0000.5142), and all participants signed an informed consent form. This study was reported in accordance with the STROBE Statement guidelines.

Inclusion and exclusion criteria: Participants were included if they were 60 years of age or older, both sexes, and lived in the community. They were excluded if they presented cognitive deficits screened by the Mini‐Mental State Examination [31], amputation, fractures, or surgery of the lower limbs in the last 6 months; or had the presence of disease or sequelae that prevented them from performing the tests.

**FIGURE 1:**
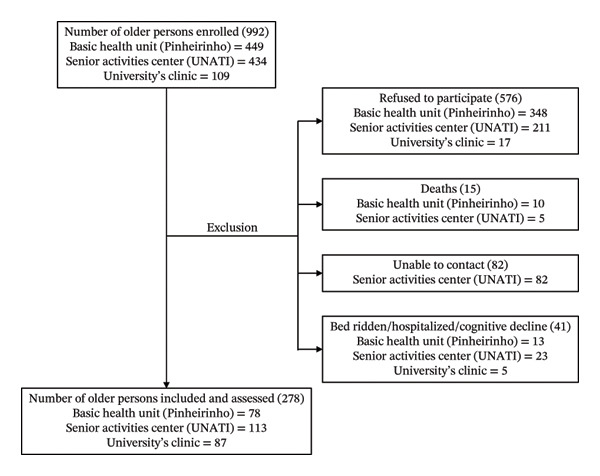
Flow diagram of participants contacted, reasons for exclusion and included in the final analysis.

Sample: Sample size was calculated using formula 10 (K+1), where *k* is the number of independent variables, according to Peduzzi et al. [32]. Considering 6 variables used in the logistic regression model, 70 subjects was the minimum sample recommended. The participant selection process is detailed in Figure 1. The present report constitutes an analysis of data from 278 participants who underwent an interview regarding sociodemographic data, information about previous falls in the last 12 months, life habits, and clinical conditions in a structured questionnaire developed by the researchers. Participants also were subject to the Physiological Profile Assessment (PPA). Trained examiners conducted interviews and tests.

### 2.1. Instruments and Measures

#### 2.1.1. Sociodemographic Characteristics

The variables included age, gender, and education. Age was presented in years and gender was categorized as male or female. Education level was expressed in years of formal schooling.

#### 2.1.2. Health‐Related Characteristics

This section included use of medication, chronic conditions, and subjective health status. Participants reported the medications they took on a regular basis, and the quantity was recorded. Regarding chronic conditions, participants were asked if any doctor has ever said that they presented any of the following conditions: hypertension, diabetes, Parkinson’s disease, depression, vertigo, incontinence, osteoporosis, arthritis or rheumatism, osteoarthritis, heart disease (angina, arrhythmia, and cardiac insufficiency), lung disease (OCPD and asthma), or others—to which participants answered “yes” or “no”; the total number of conditions was recorded. Subjective health was based on the question “In general, how do you perceive your health?” and possible answers were “good”, “average”, or “poor”.

#### 2.1.3. Depressive Symptoms

This section is assessed with the Geriatric Depression Scale (GDS) with 15 items in its translated and adapted version for the Brazilian population [33]. The cut‐off point considered as positive screening for depression is 6 or more points [34].

#### 2.1.4. Fall Risk

This section is assessed by the PPA in its short version [35]. The instrument was developed considering the main physiological factors that contribute to postural stability using independent tests. The results are compiled in software (Fallscreen) that provides a value for fall risk based on a logarithm and adjusted by age and sex. The use of PPA is well established in the literature for the assessment of older people with various health conditions [36] and presents good reliability for Brazilian older adults [37]. The tests are as follows: reaction time using a light stimulus attached to an adapted computer mouse where the subject presses the button as soon as the light turns on; quadriceps strength using a dynamometer attached to a chair and the participant’s ankle; proprioception where the participant is asked to touch both feet on each side of an acrylic panel; vision is assessed with the Melbourne Edge Test; and postural sway is measured with a sway meter attached to the participant’s waist that records the displacement while the participant stands on a foam mat with eyes open [35]. The composite score of all tests was used, as well as the results of each test separately. Participants were categorized for falls risk, namely: “marked”, “moderate”, “mild”, “low”, and “very low”, according to the equipment [35]. The last two categories were grouped as one due to the reduced number of participants who were categorized as such.

#### 2.1.5. Characteristics of Falls in the Last 12 Months

Obtained by self‐report [38], researchers explained the concept of falls to minimize confusion and reduce recall bias. Number of falls were categorized as none, single fall, or recurrent (if there were 2 or more). Participants were asked whether they had any limitations in daily activities due to falling, the occurrence of fractures, or injury related to falling and where the fall(s) took place—inside or outside the house.

Statistical analysis: tests were performed on SPSS (for Windows, version 23.0), Stata (for Windows, Version 16.0), and R (Version 4.1.6). Sociodemographic data are presented using central tendency measures and relative frequency. Groups of individuals who reported falling in the last 12 months and the ones who did not fall were compared for each of the sociodemographic variables, including depressive symptoms, using the Mann–Whitney test for continuous data and the chi‐squared test for categorical data.

Next, unadjusted and adjusted by sex, age, number of morbidities, number of medications, and subjective health status binomial logistic regression models were conducted to estimate odds ratios (ORs) to examine the relation between GDS score and the occurrence of falls. Akaike’s Information Criterion (AIC) and the likelihood ratio chi‐squared test were used to examine the goodness‐of‐fit of the model.

Further analysis was conducted using a Poisson regression model to verify the association of depressive symptoms and the number of falls. Poisson regression is used to project the number of times an event may occur in a time interval (in this case, fall in the last 12 months); however, it has to fulfill a couple of preconditions: the events have to be independent and equally dispersed. Thus, an overdispersion check was performed once the mean number of falls in the last 12 months was 0.63 and the standard deviation 1.29. A value of 1.58 was found, indicating overdispersion, and a negative binomial model was adjusted. Poisson’s model AIC was 667.26 and the negative binomial model 594.37, indicating better adjustment in the latter. Due to the number of zeroes of the dependent variable, as a sensitivity analysis, a hurdle model was used, considering the perspective that the person who has fallen once has a higher chance of falling one more time [39]. This model consists of dividing the sample into two groups: fallers (those who reported falling at least once in the last 12 months), and nonfallers (those who have not fallen) to estimate the prevalence ratio (PR). The AIC found for this model was 593.66, showing an irrelevant difference compared to the negative binomial model; therefore, the latter was considered in the results. For the negative binomial model, marginal effects of each independent variable were also calculated, assessing the effect of each one on the absolute number of falls in the past year.

Regarding the regression models, the assumptions of Events per Variable (EPV) and Variance Inflation Factor (VIF) were evaluated. For EPV, 98 individuals experienced the event, and the adjusted model included 6 independent variables; thus, 98/6 = 16.3, which is greater than 10 and therefore adequate. For VIF, values ranged from 1.02 to 1.52, all below 5, indicating no issues with multicollinearity in any of the models.

Linearity for continuous variables in the logistic regression model was assessed using the inclusion of quadratic terms. All terms showed *p* > 0.05, indicating no evidence of deviation from linearity for these variables.

Groups of individuals with positive and negative screening for depressive symptoms were compared regarding the risk of falls with the composite PPA score with the Mann–Whitney test. The same procedure was adopted for each of the PPA tests separately to verify which of the physiological components might be compromised by depressive symptoms. The Spearman’s test was conducted to examine the correlation between GDS score and PPA composite score and for each test separately.

Statistical significance was considered with *p* values under 5%. No missing data were observed for the variables included in the analyses.

## 3. Results

A total of 278 participants were included, with ages ranging from 60 to 89 years old. The participants were mostly women and did not complete high school in years of formal education. Ninety‐eight (35.3%) participants reported falling in the last 12 months, and 34 of those were recurrent fallers (constituting those who reported more than two falls in the last year). Characteristics of the sample are presented in Table 1 with comparisons between groups of participants who did not fall and those who reported falling in the last year regarding each of the variables. There was a statistically significant difference in the number of medications and chronic health conditions between the groups, as well as in subjective health status.

**TABLE 1:** Demographic and clinical characteristics of the participants.

		**Overall**	**Fallers**	**Nonfallers**	**p** **value**

Age (years), mean (SD)		70.17 (6.26)	70.59 (6.69)	70.03 (6.16)	NS^a^
Sex (female), *n* (%)		188 (67.60)	72 (73.47)	116 (64.40)	NS^b^
Education (years), mean (SD)		5.97 (4.64)	6.14 (4.93)	5.35 (4.52)	NS^a^
Number of morbidities, mean (SD)		3.59 (2.24)	4.01 (2.17)	3.37 (2.19)	**0.013** ^a^
Number of medications, mean (SD)		3.82 (2.86)	3.91 (2.89)	3.25 (2.89)	**0.042** ^a^
GDS, mean (SD)		3.73 (3.02)	4.32 (3.35)	3.42 (2.84)	**0.039** ^a^
Scores < 6, *n* (%)		208 (74.80)	67 (69.07)	141 (78.33)	NS^b^
Scores ≥ 6, *n* (%)		70 (25.20)	30 (30.93)	39 (21.67)	
Subjective health status	Good, *n* (%)	152 (54.70)	44 (44.90)	108 (60.00)	**0.007** ^b^
	Average, *n* (%)	103 (37.10)	40 (40.82)	63 (35.00)	
	Poor, *n* (%)	23 (8.3)	14 (14.29)	9 (5.00)	

PPA, composite score, mean (SD)		2.38 (1.61)	2.72 (1.87)	2.24 (1.42)	NS^a^
PPA, categories	Marked, *n* (%)	151 (54.30)	59 (60.20)	92 (51.11)	NS^b^
	Moderate, *n* (%)	74 (26.60)	21 (21.43)	53 (29.44)	
	Mild, *n* (%)	50 (18.00)	18 (18.37)	32 (17.78)	
	Low/very low, *n* (%)	3 (1.1)	0	3 (1.67)	

*Note:* Data are means (standard deviation ‐ SD) for continuous variables or number ‐*n* (percentage) for categorical variables. NS = nonsignificant (*p* > 0.05). Statistical bold significance was considered with *p* < 0.05.

Abbreviations: GDS = Geriatric Depression Scale, PPA = Physiological Profile Assessment.

^a^Mann–Whitney test.

^b^Chi‐squared test.

Approximately a quarter of the sample was considered positive for depression screening. There was a significant difference in GDS score between fallers and those who have not fallen in the last 12 months, as presented in Table 1. More than half of the participants (54.3%) presented a marked risk of falling according to the PPA categories, followed by 26.6% who presented moderate risk, 18% mild risk, and low and very low categories grouped together with 1.1% participants. No difference was observed in risk of falls between those who had fallen and those who had not. Further data are presented in Table 1. No difference was observed in any of the variables between single and recurrent fallers.

Unadjusted and adjusted binary logistic regression models were performed with the occurrence of falls as the dependent variable and GDS score as the independent variable. The unadjusted logistic regression model showed that individuals with higher scores in the GDS presented a higher chance of falling (OR: 1.10; 95% CI: 1.01–1.19). However, this association did not remain in the adjusted model; still, good self‐perceived health was associated with a lower chance of fall occurrence in the last 12 months (OR: 0.26; 95% CI: 0.08–0.82), as shown in Table 2. AIC in the adjusted model was 334.22 and in the unadjusted 357.32, indicating better quality of the adjusted model in the assessment for association between variables.

**TABLE 2:** Binary logistic regression model to evaluate the association between occurrence of falls and GDS score.

Occurrence of falls in the last 12 months	Odds ratio	*p* value	95% confidence interval
*Unadjusted Binary Logistic Regression Model*			
GDS score	1.10	0.05	1.01–1.19
Constant	0.37	< 0.001	—

*Adjusted Binary Logistic Regression Model*			
GDS score	1.01	0.73	0.99–1.02

*Sex*			
Female	Reference	Reference	Reference
Male	0.68	0.22	0.36–1.25
Age	1.01	0.40	0.97–1.06
Number of morbidities	1.02	0.69 0.88 – 1.18	
Number of medications	1.02	0.70 0.91 – 1.13	

*Subjective health status*			
Poor	Reference	Reference	Reference
Average	0.36	0.05	0.12–1.06
Good	0.26	0.01	0.08–0.82
Constant	0.41	< 0.01	

Abbreviation: GDS = Geriatric Depression Scale.

A binomial negative model was conducted to examine the association between number of falls and GDS score. The crude model presented no association between GDS score and number of falls (PR: 1.05; 95% CI: 0.97–1.12). Another model was conducted to confirm this association fitted by sex, age, and the variables that presented differences between fallers and nonfallers: number of chronic conditions, number of medication, and self‐reported health status. The association between GDS and occurrence of falls in the fitted model was no longer significant (PR: 0.98; 95% CI: 0.90–1.07). On the other hand, there is an association between the number of chronic conditions and the number of falls (PR: 1.12; 95% CI: 1.08–1.28). Marginal effects were observed for the number of comorbidities, indicating that older people with more comorbidities have an average increase of 0.07 falls per year. The data are presented in Table 3. A total of 28 participants reported taking antidepressant medication; however, there was no difference between groups (data not presented), probably due to the low number, thus it was not considered a confounder in the analysis.

**TABLE 3:** Negative binomial regression model to examine the association between number of falls and GDS score.

**Unadjusted coefficients (negative binomial model)**					
		**Prevalence ratio (PR)**	**95%CI**	**Marginal effects**	***p* value**

GDS score		1.05	0.97–1.12	0.03	0.17

*Adjusted Coefficients (Negative Binomial Model)*					
GDS score		0.98	0.90–1.07	−0.01	0.72
Sex	Female	Reference			
	Male	0.65	0.38–1.18	−0.27	0.10

Age		1.02	0.99–1.05	0.01	0.18
Number of morbidities		1.12**^∗^**	1.08–1.28**^∗^**	**0.07**	**0.04**
Number of medications		0.88	0.68–1.05	−0.02	0.43
Subjective health status	Poor	Reference			
	Average	0.54	0.23–1.35	−0.20	0.12
	Good	0.38	1.12–1.10	−0.43	0.35

*Note:* Statistical bold significance was considered with *p* < 0.05.

Abbreviation: GDS = Geriatric Depression Scale.

^∗^Highlights significant findings considering confidence interval of 95% (CI 95%).

There is a statistically significant difference between individuals with positive and negative screening for depressive symptoms (GDS as a dichotomous variable) in risk of falling according to the PPA. However, when the tests are analyzed separately, the only one with statistical significance between groups is reaction time. The results are displayed in Table 4.

**TABLE 4:** Group comparison between participants with positive and negative screening for depression.

	**Mann–Whitney test**	***Z***	**p** **value**

PPA score	5695.5	−2.72	0.01**^∗∗^**
Melbourne Edge Test	6326	−1.65	0.10
Proprioception	6979	−0.52	0.61
Quadriceps strength	6309	−1.67	0.10
Reaction time	5318.5	−3.37	0.001**^∗∗∗^**
Postural Sway	7247.5	−0.06	0.95

*Note:* Statistical bold significance was considered with *p* < 0.05.

Abbreviations: GDS = Geriatric Depression Scale, PPA = Physiological Profile Assessment.

^∗^ *p* < 0.05.

^∗∗^ *p* < 0.01.

^∗∗∗^ *p* < 0.001.

There was a positive correlation between GDS scores (as a continuous variable) and risk of falling, considering the PPA composite score. This correlation is found in examining each test separately in the Melbourne Edge Test, muscle strength of the quadriceps, and reaction time. The results are presented in Table 5.

**TABLE 5:** Correlation of GDS scores and PPA composite score and individual tests.

		**GDS score**

GDS score	Correlation coefficient	1
	*p* value	.

PPA score	Correlation coefficient	0.20
	*p* value	0.00^∗∗∗^

Melbourne Edge Test	Correlation coefficient	−0.16
	*p* value	0.01^∗∗^

Proprioception	Correlation coefficient	0.08
	*p* value	0.18

Quadriceps strength	Correlation coefficient	−0.17
	*p* value	0.01^∗∗^

Reaction time	Correlation coefficient	0.20
	*p* value	0.00^∗∗∗^

Postural sway	Correlation coefficient	0.03
	*p* value	0.64

*Note:* Statistical significance was considered with *p* < 0.05.

Abbreviations: GDS = Geriatric Depression Scale, PPA = Physiological Profile Assessment.

^∗^ *p* < 0.05.

^∗∗^ *p* < 0.01.

^∗∗∗^ *p* < 0.001.

## 4. Discussion

The current guideline for falls prevention and management states that all older adults should be advised on fall prevention, and those at risk should be offered a multidomain assessment to establish the appropriate intervention for each case [30]. These guidelines emphasize that fall risk in older adults results from the interaction between physical, cognitive, and psychological factors, rather than from isolated impairments, and that identifying risk factors for falls in older people enables establishing the most effective preventive strategies. Classic evidence [29] has highlighted the importance of muscle weakness, balance, and gait deficits as the most prominent individual risk factors for falls in older adults. However, the multifactorial nature of falls leads to a broader investigation of additional contributing factors, including psychological aspects. In recent years, psychological factors received increasing attention in the literature, potentially increasing the risk of falling in the older population, especially because the medication‐based treatment of such conditions is reported to be another risk factor for falls due to their side effects [26, 40–42]. Within this context, the present study provides a more detailed examination of the association between depressive symptoms and falls while accounting for established physiological risk factors.

The present study initially compares groups of fallers and nonfallers over the last 12 months, and among the characteristics of this population, there is a significant difference in the GDS score. Likewise, the literature points to an association between depressive symptomatology and the occurrence of falls in both transversal and longitudinal studies [40, 43–46], even though different instruments were used to assess depressive symptoms and the concept of depressive symptoms is not always differentiated from depression. Britton et al. in 2019 [43] found a difference in GDS scores between participants who reported single and recurrent falls when compared to those who did not fall. Jo et al. [45] and Li et al. [44] reported similar results; however, Jo used one question to indicate depression, and Li used the Patient Health Questionnaire‐2 (PHQ‐2) to categorize participants into depressed and not depressed. Likewise, Quach et al. indicate that clinically significant depressive symptoms screened with the Center for Epidemiologic Studies Depression‐Revised (CESD‐R) scale increased fall risk.

Beyond depressive symptoms, other characteristics distinguished fallers from nonfallers in the present study, such as the number of medications, self‐reported health status, and the number of morbidities reported by the participants. These findings suggest there might be other factors which could act as mediators or common risk factors for both conditions, as previously proposed by Iaboni and Flint [7]. Similar patterns have been reported in prior studies showing associations between falls and multimorbidity [43–45], polypharmacy [46], and subjective health status [45]. Together, these findings reinforce the importance of considering broader health complexity when examining fall risk in older adults.

There is an association between GDS scores and falls observed in the binary logistic regression model. There is a higher chance of presenting a fall for every point in GDS score (OR: 1.09). These findings align with proposed mechanisms whereby depressive symptoms may contribute to psychomotor slowing [9, 13], gait instability [14], and delayed reaction time [12], thereby increasing fall risk. Conversely, falls themselves may precipitate or exacerbate depressive symptoms through fear of falling, loss of confidence, functional decline, and social withdrawal [15]. Although the present study cannot establish causality, the results support the existence of a clinically meaningful association between depressive symptoms and falls.

Recurrent falls are often associated with poorer health outcomes [29, 47], in addition to increased risk of subsequent falls. In contrast to the studies presented by Britton and Jo, the negative binomial regression model presented no association between depressive symptoms and number of falls after adjusting for other variables. This discrepancy may be partially explained by differences in depressive symptom assessment tools and by variations in the prevalence of depressive symptoms across study populations. Britton used GDS with 30 items and Jo used one question, “In the past 1 year, have you felt sadness or despair to the extent that you could not complete everyday activities for 2 consecutive weeks or more?“. Another possibility to explain such a difference might be the prevalence of participants considered positive for depressive symptoms—25% in the present study, 16% in the study by Britton, and 8% by Jo. The prevalence of depressive symptomatology found in this study aligns with findings from other Brazilian studies [48, 49]. Once again, emphasizing the need to interpret international comparisons cautiously given cultural, methodological, and conceptual differences in the assessment of depressive symptoms and depression [22].

At the same time, other risk factors for falls are highlighted when adjusting the negative binomial model for sex, age, number of morbidities, subjective health status, and use of medication. Most studies are limited in assessing one condition at a time when associated with falls; however, older adults frequently experience multiple coexisting chronic conditions, which may collectively increase vulnerability to both depressive symptoms and falls. Paliwal, Slattum, and Ratliff found an association between many chronic conditions and the occurrence of a fall and recurrent falls [50]; amongst the many conditions examined, they highlight the importance of depression in the occurrence of falls. This reinforces the notion that falls prevention strategies should move beyond single‐risk‐factor models and adopt a more integrated approach that reflects the complexity of aging‐related health profiles.

Finally, this study explored specific physiological components associated with depressive symptoms. Those with depressive symptoms have slower reaction times when compared to their nondepressive counterparts, while proprioception, quadriceps strength, and postural sway are not affected. Depressive symptoms may negatively affect attention, executive function, and psychomotor speed [14, 51, 52], which are critical for generating rapid motor responses to environmental perturbations. Consequently, slower reaction time may represent a potential pathway through which depressive symptoms contribute to increased fall susceptibility. These findings suggest that reaction time may represent a particularly sensitive physiological marker linking depressive symptoms and fall risk. Although no other studies have systematically compared individual components of the PPA in this context, Kvelde et al. [12] found that retirement village residents with higher GDS scores presented slower reaction times, and that this association was mediated by quadriceps strength. Legg et al. found that upper limb strength was associated to faster response time [53], and Neri et al. found that poor handgrip strength was associated with higher risk of falls in older Brazilian women [54]. In addition, reaction time is a modifiable factor that can be targeted through multidomain interventions and should be assessed and treated as a part of individualized tailored interventions, as suggested by Montero‐Odasso [30]. There was also a correlation between muscle strength and GDS scores, even though there was no statistical difference between groups for this variable. Kvelde et al. found that slower reaction time to choice stepping was mediated by quadriceps strength in depressed individuals [12]. The observed correlation between muscle strength and GDS scores in the present study further supports this hypothesis and suggests that subtle physiological changes may contribute to fall risk even in the absence of overt strength deficits.

Depressive symptoms and depression are prevalent in aging and often go undiagnosed, despite their potential contribution to adverse outcomes such as falls. The present study can contribute as an alert to health professionals to assess depressive symptoms, particularly in the context of fall prevention. At the same time, older adults experiencing depressive symptoms in healthcare settings may be questioned by the clinicians about falls as part of routine geriatric assessment, representing a form of opportunistic case finding. This pathway may facilitate earlier recognition of individuals at higher risk of falls and support timely preventive interventions. An additional strength of this study is the use of well‐established and standardized instruments to assess both depressive symptoms and risk of falls, which enhances the reliability and comparability of the findings.

Some limitations should also be acknowledged. First, this study has a cross‐sectional design; thus, a causal relation cannot be established. Second, convenience sampling carries a risk of selection bias. Since participants were recruited based on accessibility rather than random selection, this may potentially limit the generalizability of the findings to the broader population of community‐dwelling older adults. Even though the number of individuals recruited for this study is significant for the number of variables analyzed, only a small proportion of participants reported recurrent falls in the previous 12 months, which may have limited the ability to detect associations with this specific outcome. Furthermore, the study population presented a high incidence of polypharmacy, and the majority presented a high risk of falling according to the PPA, which may limit the generalizability of the findings to the broader older adult population in Brazil. These characteristics may, in part, be due to the use of a convenience sample. Nevertheless, the main outcomes of the study—depressive symptoms and falls—are consistent with reports from other Brazilian studies, supporting the external validity of these outcomes. Given the interplay between depressive symptoms and falls is bidirectional and mediated by other factors, research evidence should be grouped together for a better understanding of such important events in older age.

## 5. Conclusion

Overall, fallers presented more depressive symptoms, morbidities, polypharmacy, and worse subjective health status than their counterparts who did not fall in the last year. Depressive symptoms did not act directly over the number of falls; nonetheless, those with positive screening for depression presented a higher risk of falling in the PPA, which may be associated to slower reaction time. Psychological symptoms that play an important role in falls should be considered when identifying risk factors for falls and when developing prevention programs in older adults at risk for falling.

## Funding

This study was supported by the Fundação de Amparo à Pesquisa do Estado de Minas Gerais, Coordenação de Aperfeiçoamento de Pessoal de Nível Superior, and Conselho Nacional de Desenvolvimento Científico e Tecnológico.

## Disclosure

This work has been previously presented as a thesis for the master’s title at Universidade Federal de Minas Gerais [55].

The version used in this research was the one translated to brazilian Portuguese, validated by Bertolucci et al. in 1994 (Bertolucci PHF, Brucki SMD, Campacci SR, Juliano Y. O Mini‐Exame do Estado Mental em uma população geral: impacto da escolaridade. Arq Neuropsiquiatr. 1994; 52(1):01‐07. doi:10.1590/s0004‐282x1994000100001).

## Ethics Statement

The study was approved by the ethics committee from Universidade Federal de Alfenas–UNIFAL–MG (CAAE: 49987915.4.0000.5142), and all participants signed an informed consent form. When the data for this research was collected in 2015, the MMSE version used did not require permission.

## Conflicts of Interest

The authors declare no conflicts of interest.

## Data Availability

The data that support the findings of this study are available from the corresponding author upon reasonable request.
